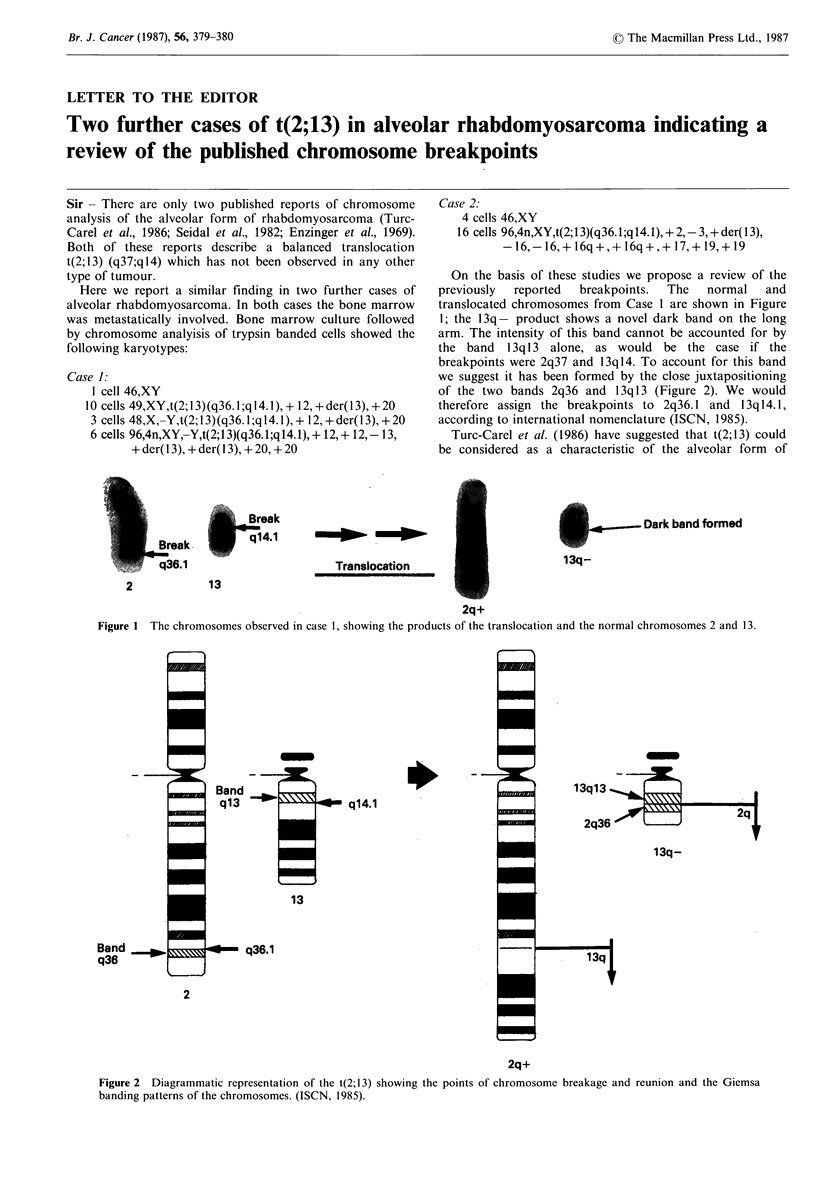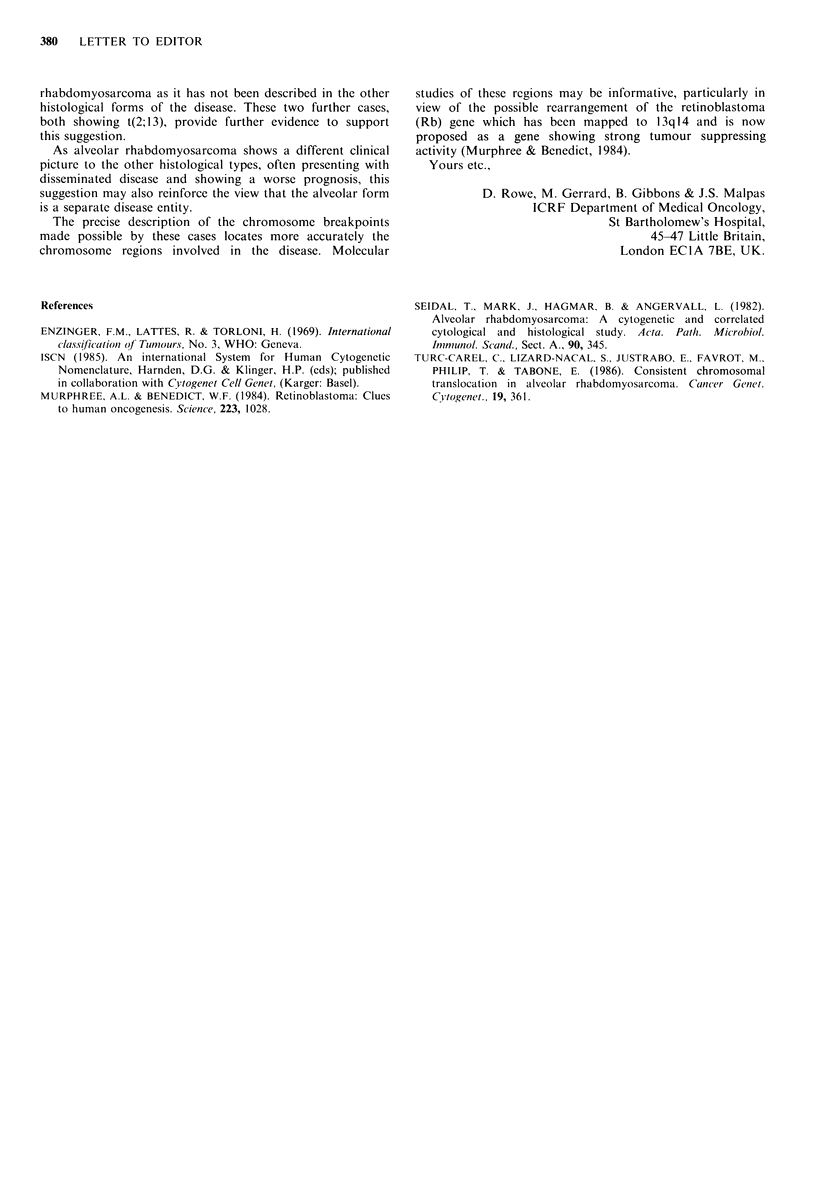# Two further cases of t(2;13) in alveolar rhabdomyosarcoma indicating a review of the published chromosome breakpoints.

**DOI:** 10.1038/bjc.1987.207

**Published:** 1987-09

**Authors:** D. Rowe, M. Gerrard, B. Gibbons, J. S. Malpas

**Affiliations:** ICRF Department of Medical Oncology, St. Bartholomew's Hospital, London, UK.

## Abstract

**Images:**


*Br. J. Cancer* (1987), **56**, 379–380 © The Macmillan Press Ltd., 1987

### LETTER TO THE EDITOR

### Two further cases of t(2;13) in alveolar rhabdomyosarcoma indicating a review of the published chromosome breakpoints

**Sir** – There are only two published reports of chromosome analysis of the alveolar form of rhabdomyosarcoma (Turc-Carel *et al.*, 1986; Seidal *et al.*, 1982; Enzinger *et al.*, 1969). Both of these reports describe a balanced translocation t(2;13) (q37;q14) which has not been observed in any other type of tumour.

Here we report a similar finding in two further cases of alveolar rhabdomyosarcoma. In both cases the bone marrow was metastatically involved. Bone marrow culture followed by chromosome analyisis of trypsin banded cells showed the following karyotypes:

*Case 1:*
1 cell 46,XY
10 cells 49,XY,t(2;13)(q36.1;q14.1),+12,+der(13),+20
3 cells 48,X,–Y,t(2;13)(q36.1;q14.1),+12,+der(13),+20
6 cells 96,4n,XY,–Y,t(2;13)(q36.1;q14.1),+12,+12,–13, +der(13),+der(13),+20,+20

*Case 2:*
4 cells 46,XY
16 cells 96,4n,XY,t(2;13)(q36.1;q14.1),+2,–3,+der(13), –16,–16,+16q+,+16q+,+17,+19,+19

On the basis of these studies we propose a review of the previously reported breakpoints. The normal and translocated chromosomes from Case 1 are shown in Figure 1; the 13q– product shows a novel dark band on the long arm. The intensity of this band cannot be accounted for by the band 13q13 alone, as would be the case if the breakpoints were 2q37 and 13q14. To account for this band we suggest it has been formed by the close juxtapositioning of the two bands 2q36 and 13q13 (Figure 2). We would therefore assign the breakpoints to 2q36.1 and 13q14.1, according to international nomenclature (ISCN, 1985).

Turc-Carel *et al.* (1986) have suggested that t(2;13) could be considered as a characteristic of the alveolar form of

**Figure 1** The chromosomes observed in case 1, showing the products of the translocation and the normal chromosomes 2 and 13.

**Figure 2** Diagrammatic representation of the t(2;13) showing the points of chromosome breakage and reunion and the Giemsa banding patterns of the chromosomes. (ISCN, 1985).

rhabdomyosarcoma as it has not been described in the other histological forms of the disease. These two further cases, both showing t(2;13), provide further evidence to support this suggestion.

As alveolar rhabdomyosarcoma shows a different clinical picture to the other histological types, often presenting with disseminated disease and showing a worse prognosis, this suggestion may also reinforce the view that the alveolar form is a separate disease entity.

The precise description of the chromosome breakpoints made possible by these cases locates more accurately the chromosome regions involved in the disease. Molecular studies of these regions may be informative, particularly in view of the possible rearrangement of the retinoblastoma (Rb) gene which has been mapped to 13q14 and is now proposed as a gene showing strong tumour suppressing activity (Murphree & Benedict, 1984).

Yours etc.,

D. Rowe, M. Gerrard, B. Gibbons & J.S. Malpas
ICRF Department of Medical Oncology,
St Bartholomew's Hospital,
45–47 Little Britain,
London EC1A 7BE, UK.

**References**

ENZINGER, F.M., LATTES, R. & TORLONI, H. (1969). *International classification of Tumours*, No. 3, WHO: Geneva.

ISCN (1985). An international System for Human Cytogenetic Nomenclature, Harnden, D.G. & Klinger, H.P. (eds); published in collaboration with *Cytogenet Cell Genet*, (Karger: Basel).

MURPHREE, A.L. & BENEDICT, W.F. (1984). Retinoblastoma: Clues to human oncogenesis. *Science*, **223**, 1028.

SEIDAL, T., MARK, J., HAGMAR, B. & ANGERVALL, L. (1982). Alveolar rhabdomyosarcoma: A cytogenetic and correlated cytological and histological study. *Acta. Path. Microbiol. Immunol. Scand.*, Sect. A., **90**, 345.

TURC-CAREL, C., LIZARD-NACAL, S., JUSTRABO, E., FAVROT, M., PHILIP, T. & TABONE, E. (1986). Consistent chromosomal translocation in alveolar rhabdomyosarcoma. *Cancer Genet. Cytogenet.*, **19**, 361.